# Cycle-Dependent Expression of Immune, Morphogenetic, Apoptotic, and Steroid-Related Markers in the Endometrium of Infertile Women: A Pilot Study

**DOI:** 10.3390/cimb48030264

**Published:** 2026-03-02

**Authors:** Elizabete Brikune, Māra Pilmane, Jana Brikune

**Affiliations:** 1 RSU Institute of Anatomy and Anthropology, Eksporta Street 1A, LV-1010 Riga, Latvia; elizabrikune@gmail.com; 2 AVA Clinic, Sabiedrība ar Ierobežotu Atbildību (Limited Liability Company), Baznīcas Street 20/22, LV-1010 Riga, Latvia

**Keywords:** endometrium, infertility, BMP-2/4, G-CSF, apoptosis, menstrual cycle

## Abstract

Infertility affects a substantial proportion of women of reproductive age and is frequently associated with impaired endometrial receptivity. Successful implantation depends on tightly regulated hormonal, immune, apoptotic, and stress-response pathways within the endometrium. This pilot study aimed to evaluate the expression and distribution of granulocyte colony-stimulating factor (G-CSF), bone morphogenetic proteins 2/4 (BMP-2/4), heat shock protein 70 (HSP-70), apoptosis, progesterone, estrogen, and pentraxin-3 (PTX-3) in the endometrium of infertile women across different menstrual cycle days. A descriptive cross-sectional analysis was performed on endometrial tissue samples obtained from six infertile women aged 21–49 years at various menstrual cycle days. Routine histology, immunohistochemistry, TUNEL assay, and chromogenic in situ hybridization were used to assess tissue morphology, protein expression, apoptotic activity, and PTX-3 gene expression. Quantitative evaluation was applied to immunohistochemical markers and apoptosis, while PTX-3 expression was assessed semi-quantitatively. G-CSF expression showed low-to-moderate levels with a relative mid-cycle increase. BMP-2/4 demonstrated the highest overall positivity across most cycle days, with marked inter-sample variability. HSP-70 exhibited pronounced cycle-dependent variability. Apoptotic activity increased toward mid-to-late cycle days. Progesterone and estrogen positivity was heterogeneous and limited to selected cycle days. PTX-3 gene expression was highest during mid-cycle days and decreased toward later phases. No clear association with patient age was observed. Conclusions: The findings indicate distinct and cycle-dependent patterns of immune, morphogenetic, apoptotic, hormonal, and inflammatory markers in the endometrium of infertile women. These results highlight the dynamic nature of endometrial regulation and suggest that altered temporal coordination of these pathways may contribute to impaired endometrial receptivity.

## 1. Introduction

Infertility is defined as the failure to conceive after 12 months of regular, unprotected sexual intercourse, with earlier evaluation recommended in women over 35 years of age or in the presence of known risk factors [1]. Its etiology is multifactorial and may involve male factors, ovulatory dysfunction, uterine abnormalities, tubal obstruction, peritoneal conditions, cervical factors, or endocrine imbalance [1]. Approximately 10–15% of women of reproductive age are affected, highlighting infertility as a significant reproductive health concern.

Successful implantation depends on precise hormonal and immune regulation of the endometrium, a dynamic tissue that undergoes cyclic proliferation, differentiation, and regression [2,3]. Coordinated interactions between estrogen, progesterone, immune mediators, apoptotic mechanisms, and morphogenetic signaling pathways are essential for decidualization and endometrial receptivity. Disruption of this balance may contribute to implantation failure and pregnancy loss [4].

Granulocyte colony-stimulating factor (G-CSF) is a hematopoietic cytokine expressed in reproductive tissues that modulates follicular maturation, implantation, angiogenesis, and immune tolerance via G-CSFR-mediated pathways [5,6]. It promotes regulatory T-cell activity, reduces natural killer cell cytotoxicity, and enhances endometrial receptivity. Altered G-CSF expression has been associated with recurrent spontaneous abortion, recurrent implantation failure, polycystic ovary syndrome, and thin endometrium [7].

Bone morphogenetic proteins (BMPs), particularly BMP-2 and BMP-4, are members of the transforming growth factor-β superfamily and play critical roles in decidualization and implantation [8,9]. Reduced BMP-2 signaling has been demonstrated in endometrial stromal cells from women with uterine leiomyomas, contributing to impaired receptivity [10,11,12]. BMP-4 regulates trophoblast invasion, apoptosis, macrophage polarization, and stromal differentiation [13,14,15,16,17,18], and its dysregulation has been linked to miscarriage, preeclampsia, and endometriosis.

Heat shock protein 70 (HSP-70) is a molecular chaperone involved in cellular protection, oxidative stress regulation, and apoptosis modulation [19]. Its expression varies across the menstrual cycle and supports endometrial adaptation to cyclic remodeling [20]. Both reduced and excessive HSP-70 levels have been associated with reproductive pathology, including recurrent pregnancy loss, endometriosis, and pregnancy complications [21,22].

Apoptosis is a physiological component of cyclic endometrial turnover. The Terminal deoxynucleotidyl transferase dUTP Nick-End Labeling (TUNEL) assay enables in situ detection of DNA fragmentation and is widely used to evaluate apoptotic activity in reproductive tissues [23,24,25,26,27]. Dysregulated apoptotic timing may contribute to impaired implantation and pathological endometrial remodeling.

Steroid hormone signaling is central to endometrial receptivity. Progesterone regulates decidualization and immune tolerance through progesterone receptor A and B isoforms [28,29]. Impaired progesterone responsiveness, often termed progesterone resistance, has been implicated in implantation failure and endometriosis [30,31]. Estrogen, particularly 17β-estradiol, promotes endometrial proliferation and modulates progesterone receptor expression through estrogen receptor α and β [32,33,34,35,36]. Proper temporal coordination between estrogen and progesterone signaling is essential for functional maturation of the endometrium.

Pentraxin-3 (*PTX-3*) is a long pentraxin involved in innate immunity, extracellular matrix organization, and angiogenesis. In the endometrium, *PTX-3* is locally produced in response to inflammatory and hormonal stimuli and contributes to tissue remodeling and implantation-related immune regulation [37]. Altered *PTX-3* expression has been associated with impaired implantation and inflammatory endometrial disorders.

In this study, a targeted marker panel was selected to capture key, mechanistically linked components of endometrial cyclic remodeling that are relevant to infertility and endometrial receptivity. G-CSF was included as an immune-modulatory cytokine implicated in receptivity-related immune tolerance and endometrial microenvironment regulation. BMP-2/4 was selected to reflect morphogenetic and decidualization-related signaling pathways involved in stromal differentiation and tissue remodeling. HSP-70 was chosen as a stress-response chaperone with documented roles in endometrial protection and apoptosis-related regulation under physiological and pathological conditions. *PTX-3* was assessed as a locally produced innate immune/inflammatory mediator associated with extracellular matrix organization and endometrial tissue remodeling. Estrogen and progesterone receptors were included to evaluate steroid hormone responsiveness and to support cycle-dependent interpretation of endometrial functional states. Finally, apoptosis was quantified using the TUNEL assay to provide a functional readout of tissue turnover dynamics.

Together, these markers provide a feasibility-oriented framework for hypothesis generation in a small archival cohort. The present study was therefore designed as a pilot, hypothesis-generating investigation aimed at detecting key regulatory factors potentially involved in endometrial receptivity impairment in clinically heterogeneous infertile population women. By evaluating their expression across different menstrual cycle phases, we sought to explore whether altered temporal coordination of immune, morphogenetic, apoptotic, inflammatory, and steroid hormone signaling pathways may contribute to infertility-associated endometrial dysfunction.

## 2. Materials and Methods

### 2.1. Study Subjects

We carried out a descriptive, cross-sectional analysis of selected samples. Endometrial tissue from six eligible infertile patients was examined, with specimens taken at varying points across the menstrual cycle. These samples were sourced from the archive of the Institute of Anatomy and Anthropology at Riga Stradiņš University and had originally been collected at Riga Maternity Hospital. All participants gave written informed consent to take part in the research and agreed to the publication of the resulting data. Consent was obtained from women after they received a comprehensive explanation of the study. The investigation complied with the principles of the Declaration of Helsinki, and the protocol received approval from the Riga Stradiņš University Ethics Committee (2-PĒK-4/105/2026).

### 2.2. Characteristics of Selected Patients

Six women were included in the study, with ages ranging from 21 to 49 years. Endometrial material was obtained on days 15 to 46 of the menstrual cycle. Menstrual cycle day was determined based on the patient-reported first day of the last menstrual period (LMP), as documented in clinical records at the time of biopsy. Menstrual cycle length varied among participants, ranging from 28 to 32 days with menses duration of 5–7 days. Two women had no history of childbirth (nulliparous), whereas four women had delivered previously (Partus): two had one previous delivery, and two had two deliveries. The number of abortions ranged from zero to two across the cohort.

Menarche occurred between 12 and 15 years of age. Three women were diagnosed with uterine myoma, of whom one exhibited concomitant adenomyosis and metrorrhagia, and another had menometrorrhagia. Two women presented with endometrial hyperplasia, one of these also experiencing metrorrhagia. One woman had ovarian dysfunction manifested clinically with metrorrhagia. A shared characteristic among all participants was infertility. All detailed clinical characteristics of the infertile women included in the study are summarized in Table 1.

Due to ethical and practical considerations, endometrial tissue from healthy women or from women with confirmed normal early pregnancy was not available for comparison. Collection of such samples would require invasive endometrial biopsy in the absence of medical indication, which is ethically unjustifiable in otherwise healthy individuals. Similarly, sampling during early normal pregnancy would pose potential risks to the ongoing gestation and is therefore not ethically permissible for purely research purposes. Consequently, access to physiologically normal endometrial tissue at precisely defined cycle phases remains limited in human studies.

For these reasons, the present investigation was restricted to clinically indicated endometrial biopsies obtained from infertile patients. The study was designed as a descriptive pilot analysis aimed at characterizing temporal expression patterns rather than establishing case–control comparisons. Patients were not stratified into subgroups according to age, obstetric history, or associated gynecological conditions. Instead, samples were evaluated longitudinally according to menstrual cycle day to explore cycle-dependent variability. Each biopsy represented an independent biological specimen from a different individual, reflecting real-world clinical heterogeneity within the infertile population.

### 2.3. Sample Collection

Following endometrial curettage, endometrial tissue samples were obtained from six selected women. Tissue was excised to include all endometrial layers, with each sample measuring no more than 2.2 cm in diameter and 2.2 mm in thickness. Fixation was carried out to preserve and stabilize tissue morphology. Specimens were first placed in 10% buffered formalin for approximately 2 h at room temperature, followed by an additional 2–4 h at 39–41 °C in a thermostat.

The laboratory utilized Stefanini’s fixative solution, consisting of formaldehyde, buffer, and picric acid, after which the material was rinsed in Tyrode’s solution. Immediately after sampling, the tissues were transferred to the Institute of Anatomy and Anthropology at Riga Stradiņš University for further processing.

### 2.4. Routine Staining

Following fixation, tissue samples were dehydrated through a graded ethanol series (70–96%), then infiltrated with paraffin. Embedding was carried out using standardized paraffin-embedding procedures with paraffin volume controlled by a dispenser. Paraffin blocks were sectioned using a Leica microtome to obtain sections 3–5 µm in thickness.

The sections underwent deparaffinization in xylene for approximately 5–15 min, followed by rehydration through descending ethanol concentrations (96–70%) for 3 min each. Routine hematoxylin–eosin (H&E) staining was then performed, with hematoxylin application for 1–10 min and eosin for 30 s to 1 min. This was followed by differentiation and clarification using a carboxylic acid–xylene sequence (1–2 min; xylene 1:3). Finally, stained sections were cover slipped for subsequent histological evaluation.

### 2.5. Immunohistochemical (IHC) Analysis

Immunohistochemical staining was performed using standard streptavidin–biotin immunostaining procedures to prepare endometrial tissue sections for analysis [38]. Five different targets were examined using the following:

- G-CSF (sc-53292, monoclonal, working dilution 1:100, Santa Cruz biotechnology, Inc., Dallas, TX, USA) [39,40];
- BMP-2/4 (AV103, monoclonal, working dilution 1:100, R&D Systems, Bio-Techne, Minneapolis, MI, USA) [41];
- HSP-70 (585054A, monoclonal, working dilution 1:50, Invitrogen, Waltham, MA, USA) [42,43];
- Progesterone (IR068, monoclonal, working dilution 1:100, Dako, Carpinteria, CA, USA) [44];
- Estrogen (GA084, monoclonal, working dilution 1:100, Dako) [45,46].

Primary antibodies were diluted using an antibody diluent (code 938B-05; Cell Marque™, Rocklin, CA, USA) to obtain the required working concentrations. The HiDef Detection™ HRP Polymer System (catalogue no: 954D-30; Cell Marque, Rocklin, CA, USA) was used for subsequent immunodetection steps.

Tissue sections were cut at approximately 3 µm thickness and completely dried before processing. Deparaffinization and rehydration were followed by incubation with the primary antibody according to manufacturer recommendations. Slides were then rinsed in IHC wash buffer and incubated with the HiDef Detection™ Amplifier (code 954D-31; Cell Marque™, Rocklin, CA, USA) for 10 min at room temperature, followed by an additional wash. Subsequently, HiDef Detection™ HRP Polymer Detector (code 954D-32; Cell Marque™, Rocklin, CA, USA) was applied for 10 min at room temperature and rinsed with IHC wash buffer.

Visualization was achieved using an HRP-compatible chromogen, applied according to manufacturer’s instructions, after which sections were rinsed with distilled or deionized water. Slides were then counterstained and cover-slipped for further microscopic evaluation. Negative controls were prepared by omitting the primary antibody during the staining procedure.

### 2.6. TUNEL

The terminal deoxynucleotidyl transferase dUTP nick-end labeling (TUNEL) assay (in situ cell death detection kit, POD, 11684817910, Roche, Germany) is a technique used to identify apoptotic cells by detecting extensive DNA fragmentation characteristic of the late phase of apoptosis [47].

### 2.7. Chromogenic In Situ Hybridization (CISH) Analysis of PTX-3

Pentraxin-3 (PTX-3) gene expression in endometrial tissue samples was evaluated using CISH. This method enables localization of specific nucleic acid sequences directly within tissue architecture, allowing for simultaneous assessment of gene expression and histomorphology.

Formalin-fixed, paraffin-embedded endometrial tissue sections were deparaffinized, rehydrated, and subjected to proteolytic digestion followed by heat-induced target retrieval using EDTA pretreatment solution.

After dehydration and air-drying, tissue sections were incubated with a PTX-3-specific probe (3q25.32, CHR3, Empire Genomics corp., Williamsville, NY, USA, 14221), and denatured at 75 °C, followed by overnight hybridization at 37 °C. Post-hybridization washes were performed using Tris-buffered saline (TBS) at controlled temperatures. Hybridized probes were detected using mouse anti-DIG antibodies and an HRP-polymer detection system. Signal visualization was achieved with diaminobenzidine (DAB), producing distinct chromogenic dot-like signals. Nuclear counterstaining was performed using Nuclear Blue solution. Slides were dehydrated, cleared, and mounted using an alcoholic mounting medium [48]. Appropriate negative controls were performed by omitting the PTX-3 probe to confirm staining specificity.

### 2.8. Assessment of Local Tissue Defense Factor Quantity

Quantitative and semi-quantitative evaluation of local tissue defense factors was performed independently by two experienced morphologists. Quantitative analysis was applied to apoptosis, G-CSF, BMP-2/4, HSP-70, progesterone-positive cells, and estrogen-positive cells. For these markers, positively stained cells were counted in three representative high-power visual fields from each endometrial tissue section using light microscopy. All quantitative assessments were performed in relation to the corresponding menstrual cycle day on which the tissue sample was obtained. Mean values and standard deviations (SDs) were calculated for each marker.

Semi-quantitative analysis was applied to PTX-3 gene expression, which was assessed using CISH. The relative abundance of PTX-3 hybridization signals was evaluated using a semi-quantitative scoring system and is summarized in Table 2 [49].

### 2.9. Statistical Analysis

Statistical analysis was conducted using IBM SPSS Statistics for Windows, Version 31.0 (IBM Corp., Armonk, NY, USA).

To evaluate relationships between marker expression levels, Spearman’s rank correlation coefficient (Spearman’s rho, rs) was calculated [50]. Correlation analysis was performed between immune (G-CSF), morphogenetic (BMP-2/4), stress-response (HSP-70), apoptotic (TUNEL), steroid-related (progesterone and estrogen receptor), and inflammatory (*PTX-3*) markers. A *p*-value < 0.05 was considered statistically significant.

The strength of correlations was interpreted as follows: rs = 0.00–0.24 (very weak), rs = 0.25–0.49 (weak), rs = 0.50–0.74 (strong), and rs = 0.75–1.00 (very strong).

## 3. Results

### 3.1. Routine Staining

**Figure 1:**
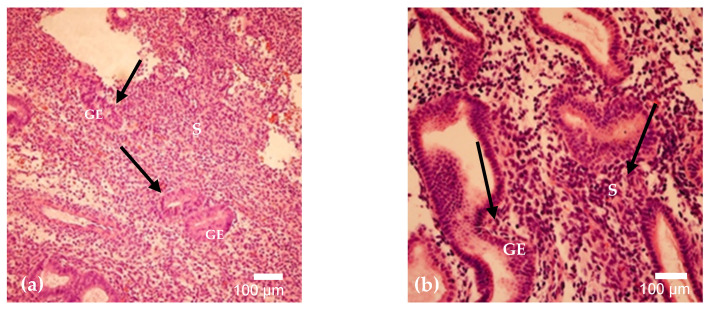
(**a**,**b**). Hematoxylin and eosin staining of endometrial tissue on cycle day 15 (**a**) and day 24 (**b**). Day 15 shows irregular tubular glands within cellular stroma with inflammatory infiltration (arrows). Day 24 demonstrates gland crowding with periglandular stromal condensation and focal inflammatory cells (arrows). Magnification: 200×. Abbreviations: GE—Glandular Epithelium; S–Stroma.

The examined endometrial samples demonstrated comparable morphological features, characterized by numerous irregularly shaped tubular glands embedded within a densely cellular stroma (Figure 1a,b). The stroma appeared markedly hypercellular, composed predominantly of compact spindle-shaped cells with scattered inflammatory infiltrates. Several glands exhibited variable luminal contours and focal crowding, while others retained relatively preserved architecture. In certain areas, periglandular stromal condensation was noted. Overall, these findings are consistent with an active/proliferative endometrial pattern with focal stromal inflammation.

### 3.2. G-CSF and BMP-2/4

**Figure 2:**
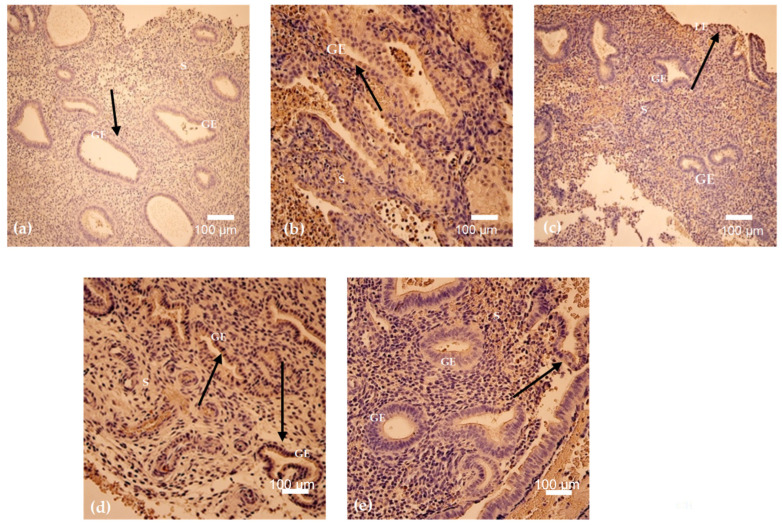
(**a**–**e**). Immunohistochemical staining for G-CSF in endometrium on cycle days 29 (**a**), 46 (**b**), 15 (**c**), 24 (**d**), and 16 (**e**). G-CSF positivity is detected mainly in stromal and periglandular cells (arrows), with focal epithelial immunoreactivity. Intensity and distribution vary between cycle days. Magnification: 200× (**a**,**c**,**d**); 250× (**b**,**e**). Abbreviations: LE—Luminal Epithelium; GE—Glandular Epithelium; S—Stroma.

**Figure 3:**
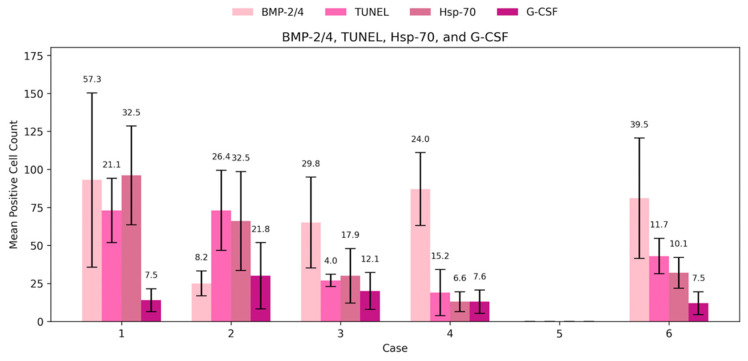
Mean positive cell count of Hsp-70, TUNEL, G-CSF and BMP-2/4.

G-CSF expression demonstrated cycle-dependent variability, with a relative increase during the mid-cycle period and lower levels at later menstrual cycle days. G-CSF-positive cells were detected in all analyzed endometrial samples except the specimen obtained on cycle day 23, in which no positivity was observed (Figure 2a–e and Figure 3). The highest mean G-CSF positivity was identified on cycle day 24 (mean = 30, SD = 21.78), reflecting marked intra-sample variability. Moderate expression was observed on cycle day 15 (mean = 20, SD = 12.12), while samples obtained on cycle days 16 and 46 demonstrated low to moderate positivity with comparable mean values. The lowest detectable G-CSF positivity among positive samples was recorded on cycle day 29 (mean = 12, SD = 7.55). Overall, G-CSF expression across the menstrual cycle was predominantly low to moderate, with a single peak on cycle day 24, indicating substantial variability in distribution (Table 3).

**Figure 4:**
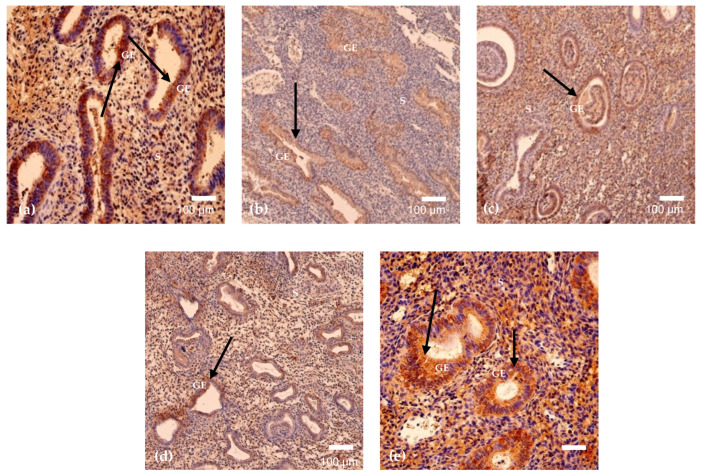
(**a**–**e**). BMP-2/4 immunostaining in endometrium on cycle days 29 (**a**), 46 (**b**), 15 (**c**), 24 (**d**), and 16 (**e**). Widespread cytoplasmic positivity is detected in glandular epithelium and stroma, with cycle-dependent variability in intensity and distribution (arrows). Periglandular and perivascular stromal staining is present in several samples. Magnification: 250× (**a**,**e**); 200× (**b**–**d**). Abbreviations: GE—Glandular Epithelium; S—Stroma.

BMP-2/4 expression was detected in five out of six analyzed menstrual cycle days and exhibited the highest overall levels among the evaluated markers. No BMP-2/4-positive cells were observed in the sample obtained on cycle day 23 (Figure 3 and Figure 4a–e). The highest mean BMP-2/4 positivity was recorded on cycle day 46 (mean = 93, SD = 57.33), with similarly elevated expression detected on cycle day 16. Moderate positivity was observed on cycle days 29 and 15, whereas the lowest expression among positive samples was identified on cycle day 24 (mean = 25, SD = 8.19). Overall, BMP-2/4 expression demonstrated pronounced quantitative variability across the menstrual cycle, with absence of detectable expression on cycle day 23 (Table 3).

### 3.3. HSP-70 and Apoptosis

**Figure 5:**
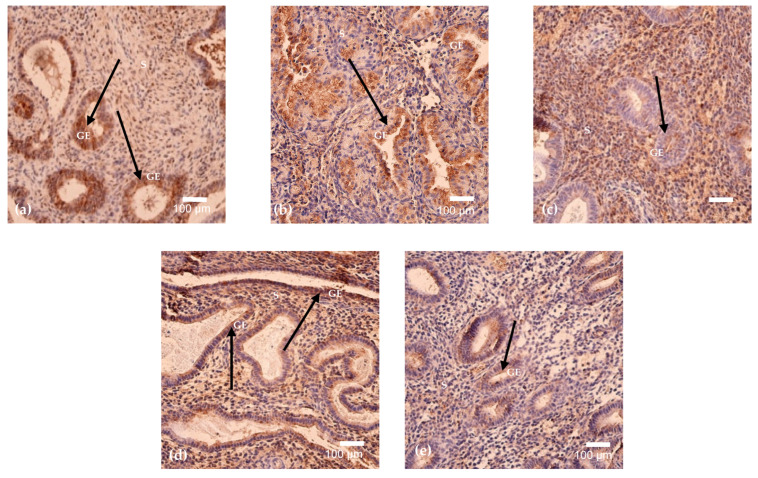
(**a**–**e**). HSP-70 immunostaining in endometrium on cycle days 29 (**a**), 46 (**b**), 15 (**c**), 24 (**d**), and 16 (**e**). Cytoplasmic positivity is detected in glandular epithelial and stromal cells, demonstrating marked cycle-dependent variability (arrows), with reduced expression on day 16. Magnification: 250× (**a**,**c**,**d**); 200× (**b**,**e**). Abbreviations: GE—Glandular Epithelium; S—Stroma.

Heat shock protein 70 (HSP-70) expression demonstrated the most pronounced variability across menstrual cycle days among the analyzed markers. HSP-70-positive cells were detected in endometrial samples obtained on five out of six cycle days, with no detectable positivity in the specimen obtained on cycle day 23 (Figure 3 and Figure 5a–e). The highest mean HSP-70 positivity was observed on cycle day 46 (mean = 96, SD = 32.52), while relatively elevated expression was also recorded on cycle day 24 (mean = 66, SD = 32.53). Moderate positivity was identified on cycle days 29 and 15, with mean values of 32 and 30, respectively. The lowest detectable HSP-70 positivity among positive samples was observed on cycle day 16 (mean = 13, SD = 6.56). Overall, HSP-70 expression was present across multiple menstrual cycle days and exhibited marked quantitative variability, with complete absence of detectable expression on cycle day 23 (Table 3).

**Figure 6:**
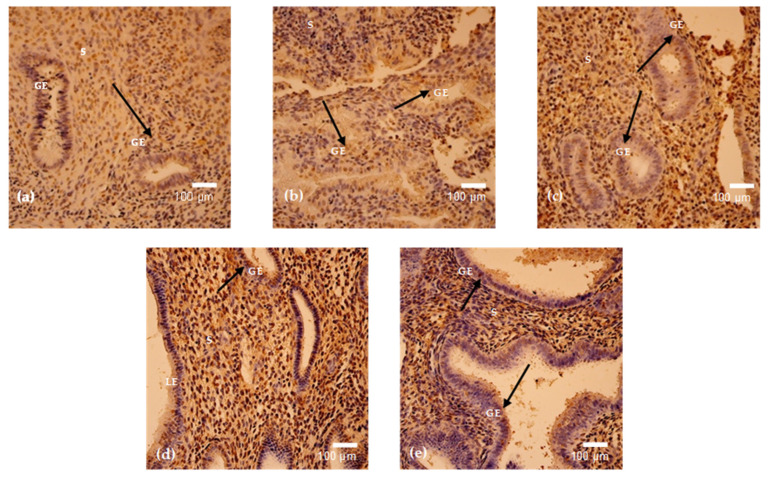
(**a**–**e**). Detection of apoptosis in endometrial tissue by TUNEL staining on cycle days 29 (**a**), 46 (**b**), 15 (c), 24 (**d**), and 16 (**e**). Positive nuclei are identified in glandular and stromal regions (arrows), demonstrating cycle-dependent variability in distribution and intensity. Magnification: 200× (**a**–**c**); 250× (**d**,**e**). Abbreviations: LE—Luminal Epithelium; GE—Glandular Epithelium; S—Stroma.

Apoptotic activity, assessed using TUNEL staining, was detected in five out of six analyzed menstrual cycle days and was absent in the sample obtained on cycle day 23 (Figure 3 and Figure 6a–e). The highest mean apoptotic activity was observed on cycle days 46 and 24, both demonstrating a mean of 73 positive cells (SD = 21.12 and SD = 26.36, respectively), indicating substantial variability. Moderate apoptotic activity was recorded on cycle day 29, whereas lower TUNEL positivity was observed on cycle days 15 and 16, with mean values of 27 and 19, respectively. Overall, apoptotic activity demonstrated inter-sample variability across the menstrual cycle, with absence of detectable positivity on cycle day 23 (Table 3).

### 3.4. Progesterone and Estrogen

**Figure 7:**
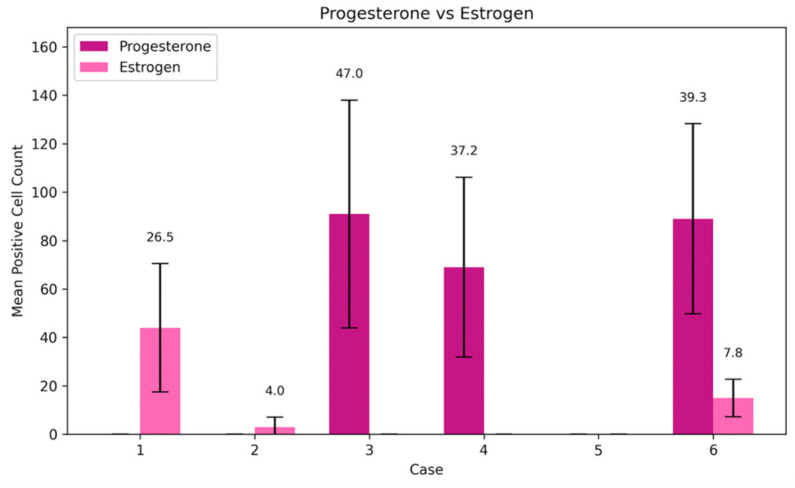
Mean positive cell count of progesterone and estrogen.

**Figure 8:**
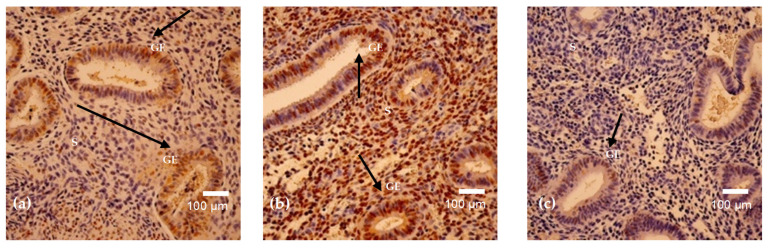
(**a**–**c**). Progesterone receptor immunostaining in endometrium on cycle days 29 (**a**), 15 (**b**), and 16 (**c**). Cytoplasmic positivity is detected mainly in glandular epithelial cells (arrows), with heterogeneous stromal staining. Magnification: 250×. Abbreviations: GE—Glandular Epithelium; S—Stroma.

Progesterone receptor positivity was detected in three out of six analyzed menstrual cycle days and demonstrated marked variability across samples (Figure 7 and Figure 8a–c). The highest mean progesterone positivity was observed on cycle day 15 (mean = 91, SD = 47.05), reflecting considerable intra-sample variation. A similarly elevated level of progesterone-positive cells was identified on cycle day 29, also demonstrating heterogeneous distribution across examined fields. Moderate progesterone positivity was recorded on cycle day 16 (mean = 69, SD = 37.17). In contrast, no progesterone-positive cells were detected in samples obtained on cycle days 23, 24, and 46. Overall, progesterone expression exhibited substantial inter-cycle variability, with clear differences between samples showing pronounced positivity and those with complete absence of detectable staining (Table 4).

**Figure 9:**
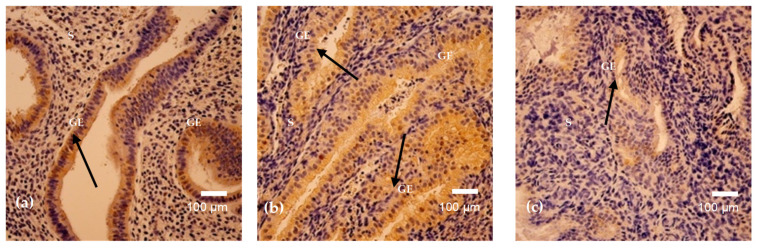
(**a**–**c**). Estrogen receptor immunostaining in endometrium on cycle days 29 (**a**), 46 (**b**), and 24 (**c**). Predominantly glandular cytoplasmic positivity is observed (arrows), with sparse stromal reactivity and cycle-dependent variation in intensity. Magnification: 250× (**a**); 200× (**b**,**c**). Abbreviations: GE—Glandular Epithelium; S—Stroma.

Estrogen receptor positivity was detected in three out of six analyzed menstrual cycle days and showed lower overall expression levels compared to progesterone (Figure 7 and Figure 9a–c). The highest mean estrogen positivity was observed on cycle day 46 (mean = 44, SD = 26.51), demonstrating moderate expression with notable intra-sample variability. Low estrogen positivity was recorded on cycle day 29 (mean = 15, SD = 7.77), while minimal expression was observed on cycle day 24 (mean = 3, SD = 4.04). No estrogen-positive cells were detected in samples obtained on cycle days 15, 16, and 23. Overall, estrogen expression was limited and variable across menstrual cycle days (Table 4).

### 3.5. PTX-3

**Figure 10:**
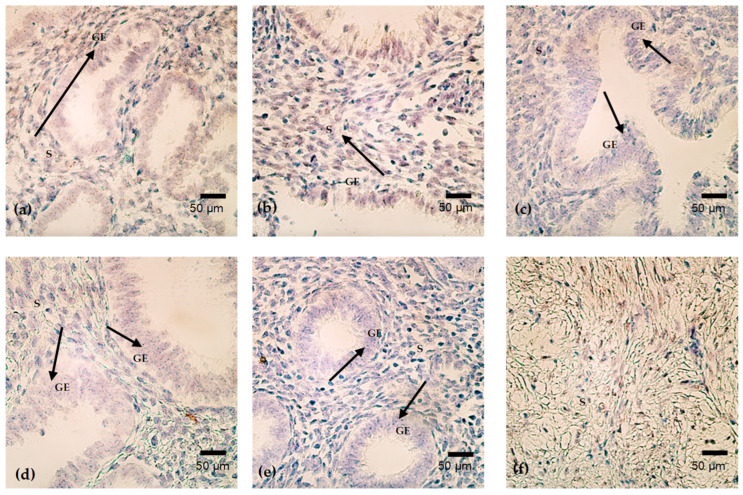
(**a**–**f**). CISH analysis of *PTX-3* gene expression in endometrium on cycle days 29 (**a**), 46 (**b**), 15 (**c**), 24 (**d**), 16 (**e**), and 23 (**f**). *PTX-3* signals are observed mainly in stromal cells, with cycle-dependent variation in epithelial involvement (arrows). Expression is absent on day 23. Magnification: 400×. Abbreviations: GE—Glandular Epithelium; S—Stroma.

*PTX-3* gene positivity demonstrated a clear association with menstrual cycle timing, with the most pronounced hybridization signals observed during the mid-cycle period and a progressive reduction toward later cycle days. Numerous *PTX-3*-positive hybridization signals (+++) were detected in endometrial samples obtained on cycle days 15 and 16, indicating a high abundance of *PTX-3* gene activity during this phase (Figure 10a–f).

In contrast, no detectable *PTX-3* gene signals were observed in the sample obtained on cycle day 23, suggesting a transient absence of measurable *PTX-3* gene expression at this time point. Moderate *PTX-3* gene positivity (++) was identified on cycle day 24, reflecting a reduced but still appreciable presence of *PTX-3* hybridization signals within the tissue.

In samples obtained on later cycle days (29 and 46), *PTX-3* gene signals were minimal and inconsistent, ranging from occasional positivity to complete absence (+/0), indicating markedly diminished *PTX-3* involvement during these phases.

Overall, *PTX-3* gene positivity across the menstrual cycle followed a mid-cycle-dominant pattern, characterized by strong signals early in the analyzed window, loss of detectable signal on cycle day 23, and only sporadic or weak positivity at later cycle days (Table 5).

### 3.6. Correlations of Studied Factors

A strong positive correlation was observed between HSP-70 expression and apoptotic activity (rs = 0.975, *p* = 0.005), indicating a close relationship between stress-response activation and apoptosis across the evaluated samples.

A perfect negative correlation was identified between progesterone and BMP-2/4 expression (rs = −1.000, *p* < 0.001). Conversely, estrogen expression demonstrated a perfect positive correlation with BMP-2/4 (rs = 1.000, *p* < 0.001). These findings reflect a strong association between steroid receptor expression patterns and BMP signaling in the evaluated subset of samples.

No statistically significant correlations were observed between G-CSF and the other analyzed markers (*p* > 0.05). Similarly, correlations between HSP-70 and BMP-2/4 (rs = 0.100, *p* = 0.873), apoptosis and BMP-2/4 (rs = −0.103, *p* = 0.870), and apoptosis and G-CSF (ρ = 0.410, *p* = 0.493) were not statistically significant.

## 4. Discussion

This pilot study examined the cycle-dependent expression of selected immune-modulatory, morphogenetic, stress-response, apoptotic, steroid hormone-related, and inflammatory markers in the infertile endometrium. Rather than identifying uniform molecular deficiencies, the findings suggest altered temporal coordination across interconnected regulatory systems. The observed heterogeneity between cycle days and individuals supports the concept that infertility-associated endometrial dysfunction may reflect dysregulated synchronization rather than absolute absence of specific factors.

Endometrial receptivity depends on tightly balanced immune modulation. Controlled inflammatory signaling is required during implantation, while excessive cytotoxic activation or inadequate immune tolerance may compromise embryo acceptance. In this context, the mid-cycle predominance of G-CSF expression observed in our cohort aligns with its documented role as a pleiotropic immunomodulatory cytokine involved in angiogenesis, immune tolerance, and tissue regeneration [51,52,53]. Experimental evidence indicates that G-CSF modulates decidual immune cell populations and suppresses cytotoxic natural killer cell activity [54,55], supporting maternal–fetal immune equilibrium. Clinical studies investigating G-CSF supplementation in implantation failure and a thin endometrium have yielded heterogeneous outcomes [56,57,58], suggesting that the biological impact of G-CSF depends not only on its presence but also on timing and local microenvironment. The temporal variability detected in our infertile cohort therefore supports the notion that mistimed immune signaling may impair receptivity even in the absence of systemic immune abnormalities.

Morphogenetic regulation, represented by BMP-2/4, demonstrated the most consistent positivity across samples. BMP signaling plays a central role in stromal differentiation, decidualization, and implantation competence [59,60,61,62]. Disruption of BMP pathways impairs uterine receptivity and fertility, while altered downstream responsiveness has been described in pathological uterine conditions [9]. The prominent BMP-2/4 positivity observed in this study may indicate preserved ligand presence; however, variability across cycle days and among individuals suggests potential differences in signaling efficiency rather than absolute expression. This observation is particularly relevant in patients with myomas or adenomyosis, where paracrine influences may induce functional resistance to morphogenetic signaling. Thus, infertility may arise not from absence of BMP expression but from altered responsiveness within a complex endocrine–immune microenvironment.

Stress response regulation, reflected by HSP-70 expression, displayed the greatest inter-sample variability. HSP-70 is a molecular chaperone essential for maintaining protein homeostasis, regulating apoptosis, and protecting cells against oxidative and inflammatory stress [63,64]. Previous investigations have demonstrated cycle-dependent modulation of heat shock proteins in endometrial tissue [19,65], emphasizing their role in cyclic remodeling. Dysregulated stress response mechanisms have been implicated in implantation failure and inflammatory uterine disorders [66]. The marked variability observed in this cohort suggests fluctuating cellular stress adaptation, potentially driven by hormonal imbalance, inflammatory signaling, or structural pathology. Importantly, both insufficient and excessive stress response activation may negatively affect implantation, indicating that optimal receptivity requires finely tuned regulation rather than maximal expression.

Apoptotic turnover represents another essential component of cyclic endometrial renewal. Increased TUNEL positivity during mid-to-late cycle days in our study corresponds to known patterns of physiological glandular regression during the late secretory phase [27,67]. Apoptotic timing is critical for maintaining structural integrity and preparing the endometrium for menstruation or implantation. Altered apoptotic synchronization has been linked to implantation failure and endometrial pathology [68,69]. The heterogeneity observed in our infertile cohort may reflect desynchronization of programmed cell death, potentially narrowing or destabilizing the implantation window. However, apoptosis was evaluated using functional DNA fragmentation assessment rather than comprehensive molecular pathway analysis, and therefore mechanistic conclusions remain preliminary.

Steroid hormone signaling orchestrates immune, morphogenetic, and apoptotic pathways. Progesterone receptor expression showed discontinuous distribution across cycle days. Given the central role of progesterone in decidualization, immune tolerance, and glandular transformation [70,71], heterogeneous receptor positivity may reflect impaired responsiveness rather than systemic hormonal insufficiency. Progesterone resistance has been widely documented in endometriosis and chronic inflammatory states [72,73,74], often occurring despite normal circulating hormone levels. Similarly, estrogen receptor expression was limited and variably distributed. Estrogen is essential for proliferative priming but must be appropriately downregulated during the receptive phase [75,76,77]. Persistent or mistimed estrogen signaling can interfere with progesterone-driven differentiation and implantation. The observed steroid receptor heterogeneity therefore supports the concept that infertility may involve dysregulated receptor dynamics rather than absolute endocrine deficiency.

*PTX-3* gene expression demonstrated a distinct mid-cycle predominance, suggesting its involvement in implantation-associated microenvironmental preparation. *PTX-3* is a multifunctional innate immune mediator implicated in extracellular matrix organization, angiogenesis, and reproductive processes [78,79,80,81]. Its expression is influenced by inflammatory cytokines, hypoxia, and steroid signaling [82,83], positioning it at the intersection of immune and hormonal regulation. The temporal pattern observed in this study reinforces the view that *PTX-3* contributes to coordinated tissue remodeling during the receptive window. Reduced expression at later cycle days may represent physiological regression; however, altered timing could compromise extracellular matrix organization and local immune modulation.

Importantly, correlation analysis further supported the interrelationship among selected regulatory pathways. A strong positive correlation was identified between HSP-70 expression and apoptotic activity, suggesting that stress-response activation may be closely associated with programmed cell turnover in the infertile endometrium. In addition, BMP-2/4 expression demonstrated strong associations with steroid receptor patterns, including a negative correlation with progesterone receptor expression and a positive correlation with estrogen receptor expression within the analyzed subset. Although these findings do not establish causal relationships, they indicate potential crosstalk between morphogenetic signaling and steroid responsiveness. Given the limited sample size and the small number of observations available for correlation analysis, these associations should be interpreted cautiously and considered hypothesis-generating.

Collectively, the findings support a model in which infertility-associated endometrial dysfunction arises from altered synchronization across immune, morphogenetic, apoptotic, stress response, and steroid-regulated pathways. BMP-2/4 exhibited the most stable presence, suggesting structural preservation of morphogenetic signaling, whereas HSP-70 and steroid receptors demonstrated the greatest variability, indicating possible instability in stress adaptation and hormonal responsiveness. Immune mediators G-CSF and *PTX-3* displayed mid-cycle predominance, further underscoring the importance of temporal coordination during implantation.

Several limitations must be considered. The small sample size, with each cycle day represented by a single case, precludes statistical generalization. Heterogeneity in patient age, reproductive history, and uterine pathology may contribute to variability. Additionally, complex biological processes were represented by selected markers rather than comprehensive molecular profiling. Furthermore, *PTX-3* was assessed at the gene expression level using chromogenic in situ hybridization, whereas other markers were evaluated at the protein level by immunohistochemistry. Because transcript levels do not necessarily directly correspond to protein abundance or biological activity, this methodological difference should be considered when interpreting comparative findings. Therefore, the present results should be interpreted as exploratory and hypothesis-generating. Future investigations incorporating fertile controls, standardized cycle dating, expanded immune and apoptotic marker panels, and integration of molecular and clinical phenotyping—including parallel transcript and protein validation—are necessary to validate and expand upon these observations.

Despite these limitations, this study provides integrative insight into the dynamic and heterogeneous regulatory landscape of infertile endometrium and highlights the importance of temporal coordination rather than isolated molecular defects in maintaining endometrial receptivity.

## 5. Conclusions

This pilot study demonstrates that immune-modulatory, morphogenetic, stress response, apoptotic, inflammatory, and steroid hormone-related markers exhibit distinct cycle-dependent variability in the endometrium of infertile women. G-CSF showed cycle-dependent variability, supporting its role in temporally regulated immune modulation of the endometrium. BMP-2/4 demonstrated the highest overall positivity, underscoring its central involvement in endometrial remodeling. HSP-70 variability reflected dynamic stress response regulation, while increased apoptosis in mid-to-late cycle days corresponded to physiological tissue turnover. Heterogeneous progesterone expression suggests altered hormonal responsiveness, whereas limited estrogen positivity emphasizes the importance of precise temporal regulation. *PTX-3* cycle-dependent expression indicates involvement in coordinated immune modulation and tissue remodeling during the receptive phase.

## Figures and Tables

**Table 1:** Info about the patients whose biopsies were obtained on different menstrual cycle days.

Case	Female Age	Day of the Cycle	Menstrual Cycle	Abortions	Partus	Menarche	Diagnosis
1.	21	46	30–32/5–6	0	0	14	Dysfunctio ovariorum; metrorrhagia; primary infertility
2.	28	24	28/6	0	0	15	Hyperplasia endometri; primary infertility
3.	34	15	30/7	0	I	13	Hyperplasia endometri; metrorrhagia; secondary infertility
4.	36	16	28/5	1	II	14	Myoma uteri; secondary infertility
5.	39	23	30/5–6	2	I	12	Myoma uteri; menometrorrhagia; secondary infertility
6.	49	29	30/5	2	II	13	Myoma uteri; adenomyosis; metrorrhagia; secondary infertility

**Table 2:** The semi-quantitative counting method and the explanation of the identifiers.

Identifier Used	Explanation
0	No staining in the visual field (0)
0/+	Occasional occurrence of positive structures in the visual field (0.5)
+	Few positive structures in the visual field (1)
++	Moderate occurrence of positive structures in the visual field (2)
+++	Numerous positive structures in the visual field (3)
++++	Abundant staining in the visual field (4)

**Table 3:** Mean ± SD of Hsp-70, apoptosis, G-CSF and BMP-2/4-positive cells on different cycle days.

Day of the Cycle	15	16	23	24	29	46
Factor						
Hsp-70	30 ± 17.95	13 ± 6.56	0	66 ± 32.53	32 ± 10.12	96 ± 32.52
BMP-2/4	65 ± 29.83	87 ± 24.03	0	25 ± 8.19	81 ± 39.54	93 ± 57.33
Apoptosis	27 ± 4.04	19 ± 15.16	0	73 ± 26.36	43 ± 11.68	73 ± 21.12
G-CSF	20 ± 12.12	13 ± 7.64	0	30 ± 21.78	12 ± 7.55	14 ± 7.55

**Table 4:** Mean ± SD of progesterone receptor and estrogen receptor-positive cells across cycle days.

Day of the Cycle	15	16	23	24	29	46
Factor						
Progesterone	91 ± 47.05	69 ± 37.17	0	0	89 ± 39.27	0
Estrogen	0	0	0	3 ± 4.04	15 ± 7.77	44 ± 26.51

**Table 5:** Semi-quantitative data analysis of *PTX-3*.

Day of the Cycle	15	16	23	24	29	46
Gene						
*PTX-3*	+++	+++	0	++	+/0	+/0

## Data Availability

The original contributions presented in this study are included in the article/Supplementary Materials. Further inquiries can be directed to the corresponding author.

## Supplementary Materials

The following supporting information can be downloaded at: https://www.mdpi.com/article/10.3390/cimb48030264/s1.
