# Supplementary material for: Cycle-Dependent Expression of Immune, Morphogenetic, Apoptotic, and Steroid-Related Markers in the Endometrium of Infertile Women: A Pilot Study
Source: Curr Issues Mol Biol. 2026 Mar 2;48(3):264. doi: 10.3390/cimb48030264 (PMC13024835; doi:10.3390/cimb48030264)

1) **Figure S1. Progesterone receptor immunohistochemistry negative control in endometrial tissue. Magnification: 250x.**

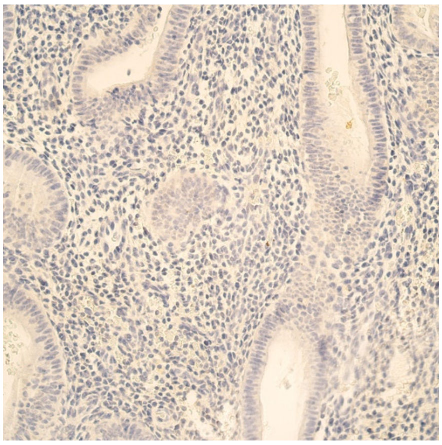

2) **Figure S2. Estrogen receptor immunohistochemistry negative control in endometrial tissue. Magnification: 250x.**

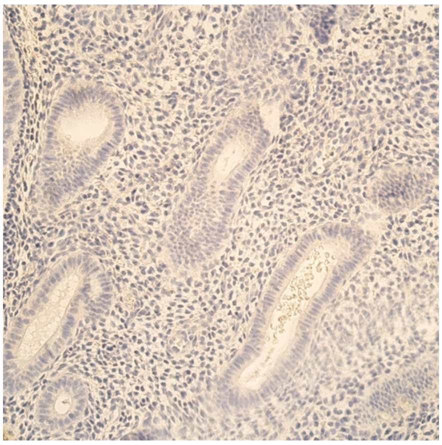

3) **Figure S3. TUNEL negative control in endometrial tissue. Magnification: 250x.**

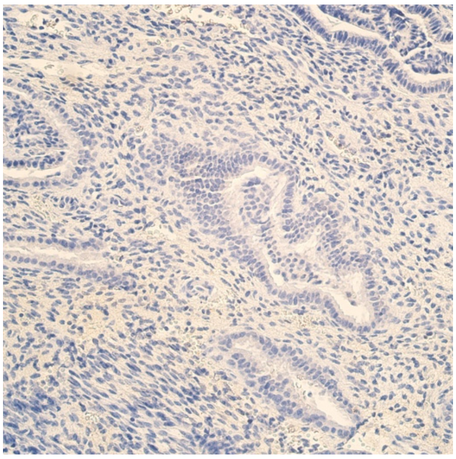

4) **Figure S4. BMP-2/4 immunohistochemistry negative control in endometrial tissue. Magnification: 250x.**

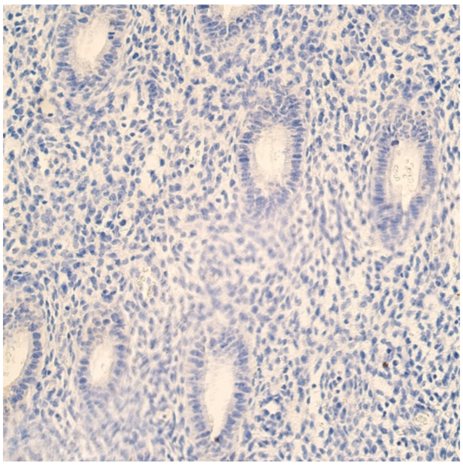

5) **Figure S5. HSP-70 immunohistochemistry negative control in endometrial tissue. Magnification: 250x.**

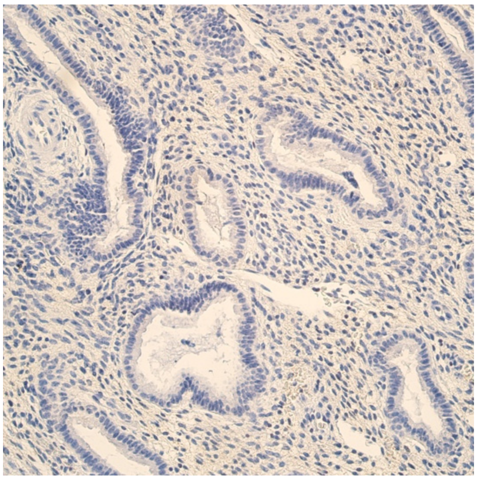

6) **Figure S6. G-CSF immunohistochemistry negative control in endometrial tissue. Magnification: 250x.**

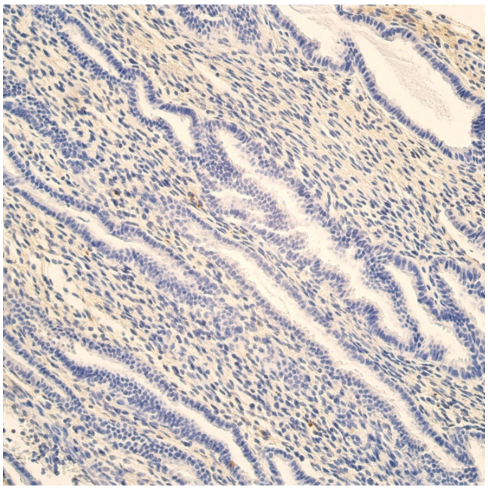

7) **Figure S7. CISH probe negative control in endometrial tissue. Magnification: 400x.**

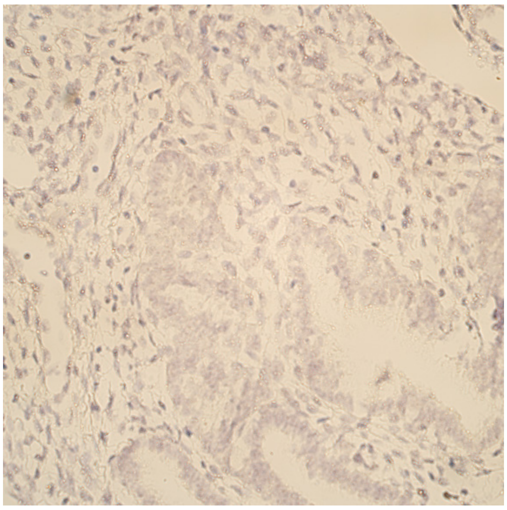

Supplement: Supplementary file 1 [file cimb-48-00264-s001.zip › cimb-4139370-supplementary.pdf]
